# Association between Statins and Retinal Vascular Occlusion: A Population-Based Cohort Study

**DOI:** 10.3390/ijerph18189864

**Published:** 2021-09-18

**Authors:** Chien-Cheng Chien, Po-Huang Chen, Chi-Hsiang Chung, Chien-An Sun, Wu-Chien Chien, Ke-Hung Chien

**Affiliations:** 1 Department of Ophthalmology, Tri-Service General Hospital, National Defense Medical Center, Taipei City 114202, Taiwan; hotjazzmichael@hotmail.com; 2 Department of Internal Medicine, Tri-Service General Hospital, National Defense Medical Center, Taipei City 114202, Taiwan; chenpohuang@hotmail.com; 3 School of Public Health, National Defense Medical Center, Taipei City 114201, Taiwan; g694810042@gmail.com; 4 Department of Medical Research, Tri-Service General Hospital, National Defense Medical Center, Taipei City 114202, Taiwan; 5 Taiwanese Injury Prevention and Safety Promotion Association, Taipei City 114201, Taiwan; 6 Department of Public Health, College of Medicine, Fu-Jen Catholic University, New Taipei City 242062, Taiwan; 040866@mail.fju.edu.tw; 7 Big Data Research Center, College of Medicine, Fu-Jen Catholic University, New Taipei City 242062, Taiwan; 8 Graduate Institute of Life Sciences, National Defense Medical Center, Taipei City 114201, Taiwan

**Keywords:** statins, retinal vascular occlusion, population-based cohort study, risk factors

## Abstract

Retinal vascular occlusion (RVO), including retinal arterial occlusion and retinal vein occlusion, is a common retinal vascular disease that causes visual disturbance. The exact pathogenesis of RVO remains unclear. In all types of RVO patients, hyperlipidemia is more than twofold more common than in controls. Statins have been used to control blood cholesterol levels and have been found to reduce the risk of cardiovascular morbidity and mortality. Moreover, the immunomodulatory functions of statins may play a role in treating inflammatory diseases. This study aimed to evaluate whether patients taking statins have a lower risk of developing RVO compared to patients not taking statins. Adult patients with statins usage on the index date identified from the Taiwan National Health Insurance Research Database (NHIRD) between 2000 and 2013 were included. A threefold matched group was selected using age, sex, and year of index date for comparison. During the mean follow-up period of 12.87 ± 1.88 years, the cumulative incidence of RVO was significantly lower in the statin-user group (29.96 per 105 person-years [PYs]) than in the non-statin-user group (39.35 per 105 PYs). The results showed a lower cumulative incidence rate of RVO in patients prescribed statins than in those not prescribed statins (log-rank test, *p* = 0.020). The adjusting hazard ratio (HR) was significantly greater for RVO in the statin-user group (adjusted HR, 0.704; 95% CI, 0.591–0.873). Statin users had a decreased risk for all types of RVO development, including central retinal artery occlusion, arterial branch occlusion, central retinal vein occlusion, and branch retinal vein occlusion. In conclusion, patients undergoing statin treatment have a lower risk of developing RVO compared to patients not taking statins.

## 1. Introduction

Retinal vascular occlusion (RVO), including retinal arterial occlusion and retinal vein occlusion, is the second most common retinal vascular disease. Retinal artery occlusion is associated with blockage of the retinal artery, often by an embolus, thrombus, or vasculitis-associated event. Retinal vein occlusion occurs when a thrombus occludes the retinal vein or secondary to atherosclerosis of the retinal artery, compressing and occluding the retinal vein [1]. Most of the cases of RVO develop at an elderly age [2]. Risk factors for RVO are diabetes mellitus (DM), hypertension (HTN), dyslipidemia [3], high body mass index, and smoking [4]. Moreover, atherosclerosis contributes to the pathophysiology of RVO [5]. Hyperlipidemia is defined as an imbalance of cholesterol levels, including low-density lipoprotein cholesterol and high-density lipoprotein cholesterol, in the blood [6]. In all types of RVO patients, hyperlipidemia is more than twofold more common than in controls [7]. Elevated serum lipid levels affect plasma viscosity and platelet function, predisposing to thrombosis and blood stasis [8]. Although the exact pathogenesis of RVO remains elusive, it is likely caused by thrombosis, which can be described by Virchow’s triad: hypercoagulability, hemodynamic changes, and endothelial injury/dysfunction [9].

RVO is the second most common form of retinal vascular disease that causes vision loss [10]. The main cause of visual impairment in RVO is macular edema, while neovascularization of the optic disc and retina are the most serious complications. Additionally, these complications lead to vitreous hemorrhage, retinal detachment, and neovascular glaucoma. Due to its multifactorial nature, RVO management remains challenging [5].

Statins, 3-hydroxy-3-methylglutaryl coenzyme A (HMG-CoA) reductase inhibitors, have been used to control blood cholesterol levels and have been found to lower cardiovascular morbidity and mortality [11,12]. The mechanism of action of statins in vascular disease is established [13,14]. More evidence of statins activities is accumulating regarding alternative immunomodulatory functions which may enhance their cardiovascular effect. Additionally, statins play a role in treating inflammatory diseases [15]. There is evidence supporting the anti-inflammatory role of statins in the pathophysiology of several eye diseases. The effects of statins have been studied in cataract [16], diabetic retinopathy [17], glaucoma [18], and age-related macular degeneration (AMD) [19].

Growth factors and inflammatory cytokines are increased in the onset of retinal ischemia [20]. This may lead to chronic inflammation [21]. In addition to their anti-inflammatory action, statins may have a protective effect on the microvascular endothelium in retinal ischemia models, thereby preventing pathological neovascularization [22]. Low doses of statins promoted vascular repair mechanisms by upregulating nitric oxide (NO) and vascular endothelial growth factor (VEGF) levels [23].

However, there is little clinical evidence evaluating the role of statins in the prevention or management of RVO. In a retrospective case–control study, no clear preventative or therapeutic benefit of statins for RVO could be found [24].

This study aimed to determine whether statins can prevent or protect against RVO, providing useful information to all physicians.

## 2. Materials and Methods

### 2.1. Data Sources

A nationwide population-based study was conducted using the Longitudinal Health Insurance Database (LHID), a subset of the National Health Insurance Research Database (NHIRD) managed by the Taiwanese National Health Research Institutions, in Taiwan from 2000 to 2013. The National Health Insurance program of Taiwan covers healthcare services for more than 99% of Taiwan’s 23 million residents [25]. Previous studies have demonstrated that this database is suitable for use in pharmacoepidemiologic research [26]. This study was approved by the Institutional Review Board of the Tri-Service General Hospital, Taipei, Taiwan (TSGH IRB No. B-110-28). To ensure confidentiality, the identifying data of all patients in the database were encrypted prior to their release for research purposes. Because the data from the NHI were deidentified, signed informed consent of the included patients was waived.

### 2.2. Inclusion and Exclusion Criteria

A retrospective cohort study of patients in the LHID was performed with a follow-up period from 1 January 2001 to 31 December 2013. We extracted data from the LHID for patients with complete sex information over 20 years of age. The statin group was defined as patients who underwent statin therapy for at least 3 months between cohort entry and index date before enrollment. A group of control patients who were threefold matched with the statin group was selected as controls, based on age (each 5-year span), sex, and index date year. Patients with or without statin use that were diagnosed with RVO prior to the index year were excluded from the analysis.

### 2.3. Study Variables

The following baseline comorbidities were identified: hypertension, diabetes mellitus, hyperlipidemia, ischemic heart disease, cerebrovascular disease, renal disease, malignancies, metabolic syndrome, hypercoagulable state, and ischemic stroke. Patients with ophthalmic diseases reported to have an impact on RVO development were enrolled, including patients with cataract [27], glaucoma [28], diabetic retinopathy [29], and age-related macular degeneration (AMD) (details of the ICD-9 codes are listed in Table S1). In addition, the urbanization level of the residency was accessible. Other considered covariates in our study were economic status, insurance cost, and the revised Charlson Comorbidity Index (CCI_R) [30], which is generally regarded as a score for the severity of systemic diseases.

### 2.4. Study Outcome

The two groups were followed until the end of 2013 to determine whether they subsequently developed RVO, including central retinal artery occlusion, arterial branch occlusion, central retinal vein occlusion, and branch retinal vein occlusion (ICD-9-CM codes, 362.31–362.32, 362.35–362.36). Subgroup analysis was conducted to ascertain the association between statin use and the incidence of different subtypes of RVO.

### 2.5. Statistical Analysis

Group differences between the statin-user group and the non-statin-user control group were analyzed using two-sample *t*-tests (for continuous variables) and chi-squared tests (for categorical variables). A survival analysis using the Kaplan–Meier estimator with a log-rank test was applied to describe and compare the cumulative hazard curves of RVO development. The Cox proportional hazard model was used to estimate the hazard ratio (HR) for the occurrence of RVO according to each variable in the univariate and multivariate analyses. SPSS 22nd edition (IBM Corp., Armonk, NY, USA) was used for all statistical analyses.

## 3. Results

### 3.1. Demographic Characteristics of the Study Sample

**Figure 1:**
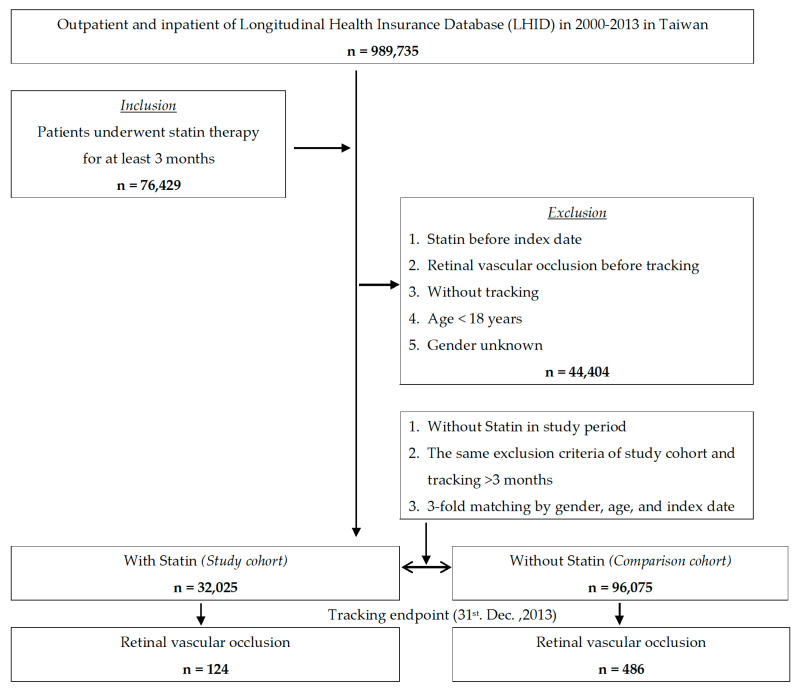
Flowchart of the study sample selection.

A total of 32,025 patients with statin use and 96,075 matched controls without statin use were enrolled in the study. Figure 1 shows the overall workflow indicating how cases and controls were drawn from the population databases, with the exclusion criteria used for both groups.

Table 1 shows the demographics of the two groups. The mean age in both groups was 52.90 ± 15.84 years. The statin-user group had a higher CCI, higher percentage of DM, higher percentage of HTN, higher percentage of hyperlipidemia, higher ischemic heart disease (IHD) percentage, higher cardiovascular disease (CVD) percentage, higher renal disease, higher malignancy, higher percentage of metabolic syndrome (MetS), higher percentage of hypercoagulable state, higher ischemic stroke rate, higher cataract rate, higher glaucoma rate, higher diabetic retinopathy rate, and higher AMD rate. During the mean follow-up period of 12.87 ± 1.88 years (details of years of follow-up in the two groups are listed in Table S2), the cumulative incidence of RVO was significantly lower in the statin-user group (29.96 per 10^5^ Person-years [PYs]) than in the non-statin-user group (39.35 per 10^5^ PYs).

### 3.2. Cumulative Hazard Curves by the Kaplan–Meier Method

**Figure 2:**
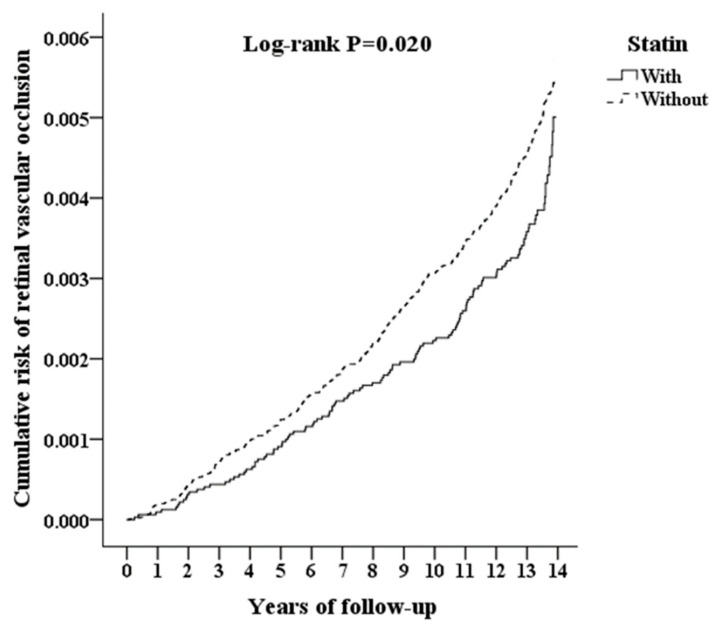
Kaplan–Meier curve for cumulative risk of retinal vascular occlusion among patients aged 18 and over stratified by statin use with a log-rank test.

The incidence of RVO during the follow-up period was illustrated using a Kaplan–Meier plot. The results showed a lower cumulative incidence rate of RVO in patients who were prescribed statins compared with those who were not prescribed statins (log-rank test, *p* = 0.020, Figure 2).

### 3.3. Univariate and Multivariate Analyses by the Cox Regression Model

The unadjusted HR for RVO was 0.792 times lower in the statin-user group than in the control group (95% confidence interval [CI], 0.650–0.965). After adjusting for covariates, a significantly greater hazard for RVO in the statin-user group remained (adjusted HR, 0.704; 95% CI, 0.591–0.873). Age was a significant risk factor for RVO development in both univariate and multivariate analyses. Patients with DM and HTN were likely to develop RVO (adjusted HR, 1.714; 95% CI, 1.512–1.888, and adjusted HR, 1.976; 95% CI, 1.501–2.630, respectively). In the multivariate analysis, patients with IHD, CVD, renal disease, MetS, and ischemic stroke had a significantly higher risk of developing RVO. Considering ophthalmic diseases, the occurrence of cataract, glaucoma, and diabetic retinopathy appeared to be risk factors with statistical significance for developing RVO in the multivariate analysis. CCI was a significant risk factor for RVO development in both univariate and multivariate analyses (crude HR, 1.234; 95% CI, 1.125–1.290; and adjusted HR, 1.200; 95% CI, 1.093–1.248) (Table 2).

### 3.4. Subgroup Analysis

We performed subgroup analyses to investigate the use of statins and the incidence of different types of RVO and found that statin users had a decreased risk for all types of RVO development, including central retinal artery occlusion (adjusted HR, 0.679; 95% CI, 0.564–0.841), arterial branch occlusion (adjusted HR, 0.620; 95% CI, 0.518–0.779), central retinal vein occlusion (adjusted HR, 0.758; 95% CI, 0.640–0.952), and branch retinal vein occlusion (adjusted HR, 0.743; 95% CI, 0.629–0.939) (Table 3).

## 4. Discussion

Our study included 32,025 Chinese patients using statins and 96,075 matched controls, with a 14-year follow-up period. We found that the cumulative incidence of all types of RVO (including central retinal artery occlusion, arterial branch occlusion, central retinal vein occlusion, and branch retinal vein occlusion) was significantly lower in the statin-user group than in the non-statin-user group (Table 3). To our knowledge, this is the first large population study focusing on the role of statins in preventing RVO. During the follow-up period, the cumulative incidence of RVO (including central retinal artery occlusion, arterial branch occlusion, central retinal vein occlusion, and branch retinal vein occlusion) was significantly lower in the statin-user group than in the non-statin-user group. In addition, the incidence of RVO during the follow-up period in the Kaplan–Meier plot showed a lower cumulative incidence rate of RVO in patients who were prescribed statins than in patients who were not prescribed statins (log-rank test, *p* value = 0.020, Figure 2). The adjusted HR for RVO was 0.704 times lower in the statin-user group than in the control group. Therefore, we found that patients receiving statin treatment have a lower risk of developing RVO compared to patients not taking statins.

The exact pathogenesis of RVO remains unclear. Inflammation has been hypothesized to play an important role in the etiology of RVO [31]. Atherosclerosis is a chronic, low-grade inflammatory condition and has been found to be related to RVO. Macrophages and T lymphocytes contribute to the initial pathology and to progress to thrombus formation later [32]. The induction of hypercoagulability is another mechanism proposed to lead to RVO. Several inflammatory chemokines/cytokines, such as tumor necrosis factor alpha, interleukin-1 beta, and interleukin-6, are prothrombogenic, being activators of the extrinsic coagulation pathway [33,34]. Moreover, local inflammation in the eye is associated with the pathogenesis of RVO. In in vivo assessment of the vitreous fluid in patients with RVO, elevated levels of proinflammatory mediators and lower levels of anti-inflammatory cytokines were found [35,36]. Patients with RVO had elevated levels of interleukin-6, interleukin-8, and monocyte chemoattractant protein-1 in a study on inflammatory immune mediators in vitreoretinal diseases [37]. Vascular endothelial growth factor was significantly elevated in patients with retinal vein occlusion [38]. All these chemokines/cytokines are considered highly proinflammatory [37,39,40]. The abovementioned evidence suggests that inflammation plays an important role in RVO.

Statins, HMG-CoA reductase inhibitors, inhibit the rate-limiting enzyme in cholesterol biosynthesis, i.e., HMG-CoA reductase [41]. Statins have been used to control blood cholesterol levels. Further, statins lower the risk of cardiovascular morbidity and mortality [11,12]. Moreover, several studies on statins have focused on the immunomodulatory effects of statins [15,23,42]. Statins exert their immunomodulatory effect not only by interfering with T cell proliferation and inhibiting the expression of costimulatory molecules on B lymphocytes [43], but also by acting as immunomodulators through the inhibition of cellular interactions and signaling molecules such as tumor necrosis factor alpha [44]. Statins can modulate the immune response by shifting the chemokine/cytokine balance from proinflammatory to anti-inflammatory [45,46].

Evidence has supported the role of inflammation in the pathophysiology of several eye diseases. The effects of statins have been researched in cataract [16], glaucoma [18], diabetic retinopathy [17], and AMD [19], with mixed results. In AMD patients, there is inadequate evidence supporting the prevention by statins of AMD or delayed disease progression [47]. Statins retarded the progression of retinopathy and improved diabetic macular edema in diabetic patients significantly [48,49]. In patients with uveitis, statins have shown a protective effect by possibly reducing uveitis development according to a retrospective population-based study [50].

However, there is little clinical evidence evaluating the role of statins in the prevention or management of RVO. A retrospective, case–control study conducted by Matei et al. analyzed 43 eyes and 129 control eyes after 43 months of follow-up in the United States. They found no clear preventative or therapeutic benefit of statins for retinal vein occlusion in high-risk patients (defined as those with hypertension and primary open-angle glaucoma) [24]. In their study, the focus was only on retinal vein occlusion with a 4-year follow-up period.

A major strength of our study is the large population (a total of 32,025 patients with statin use and 96,075 matched controls without statin use) that we examined, with a long-term follow-up (mean follow-up of 12.87 ± 1.88 years). This study used a sample from a nationwide Taiwanese population. This design reduced the selection bias, make the results applicable to the general population of Taiwan, and accurately evaluated the role of statins in RVO. To the best of our knowledge, this is the first large-size study focusing on the role of statins in the prevention of RVO.

However, the present study has a few limitations. The NHIRD database does not provide laboratory or clinical information, such as color fundus examination and fluorescein angiography for the classification of disease severity. Furthermore, statin dosage was not recorded in the NHIRD database, and it was not possible to evaluate the benefit of statins. We also did not measure the levels of cholesterol in this study. Therefore, it is uncertain whether statin usage or cholesterol is associated with RVO. Moreover, hyperlipidemia being a chronic disease, statin is prescribed to patients in Taiwan for at least 3 months with follow-up. Therefore, we defined the statin group as patients who underwent statin therapy for at least 3 months. Finally, the results are limited by the uniform ethnic background.

## 5. Conclusions

In conclusion, patients undergoing statin treatment may have a lower risk of developing RVO compared to patients not taking statins. More studies are needed to evaluate the potential benefits of statins in RVO prevention.

## Figures and Tables

**Table 1:** Characteristics of the two groups at baseline.

Statin	With		Without		*p*
Variables	*n*	%	*n*	%	
Total	32,025	25.00	96,075	75.00	
Gender					0.999
Male	15,096	47.14	45,288	47.14	
Female	16,929	52.86	50,787	52.86	
Age (years)	54.57 ± 13.51		52.34 ± 16.51		0.088
DM					<0.001 *
Without	23,513	73.42	87,195	90.76	
With	8512	26.58	8880	9.24	
HTN					<0.001 *
Without	23,600	73.69	85,693	89.19	
With	8425	26.31	10,382	10.81	
Hyperlipidemia					<0.001 *
Without	7027	21.94	95,078	98.96	
With	24,998	78.06	997	1.04	
IHD					<0.001 *
Without	29,513	92.16	91,352	95.08	
With	2512	7.84	4723	4.92	
CVD					<0.001 *
Without	28,939	90.36	90,548	94.25	
With	3086	9.64	5527	5.75	
Renal disease					<0.001 *
Without	29,180	91.12	94,770	98.64	
With	2845	8.88	1305	1.36	
Tumor					<0.001 *
Without	31,019	96.86	94,077	97.92	
With	1006	3.14	1998	2.08	
MetS					<0.001 *
Without	29,671	92.65	93,333	97.15	
With	2354	7.35	2742	2.85	
Hypercoagulable state					0.004 *
Without	31,639	98.79	95,104	98.99	
With	386	1.21	971	1.01	
Ischemic stroke					<0.001 *
Without	29,040	90.68	90,623	94.33	
With	2985	9.32	5452	5.67	
Cataract					<0.001 *
Without	30,710	95.89	92,749	96.54	
With	1315	4.11	3326	3.46	
Glaucoma					<0.001 *
Without	29,240	91.30	89,064	92.70	
With	2785	8.70	7011	7.30	
Diabetic retinopathy					<0.001 *
Without	31,243	97.56	95,084	98.97	
With	782	2.44	991	1.03	
AMD					<0.001 *
Without	31,379	97.98	94,931	98.81	
With	646	2.02	1144	1.19	
CCI	0.05 ± 0.18		0.03 ± 0.15		<0.001 *

* indicates a significant difference between the two groups with/without statin usage, *p* < 0.05. a presented as mean ± SD. CCI: Charlson comorbidity index, DM: diabetes mellitus, HTN: hypertension, IHD: ischemic heart disease, CVD: cardiovascular disease, MetS: metabolic syndrome, AMD: age-related macular degeneration.

**Table 2:** Risk factors for retinal vascular occlusion by using Cox regression.

Variables	Crude HR	95% CI	95% CI	*p*	Adjusted HR	95% CI	95% CI	*p*
Statin								
Without	Reference				Reference			
With	0.792	0.650	0.965	0.020 *	0.704	0.591	0.873	<0.001 *
Gender								
Male	0.953	0.812	1.118	0.552	0.970	0.822	1.111	0.703
Female	Reference				Reference			
Age (yrs)	1.037	1.031	1.042	<0.001 *	1.026	1.019	1.031	<0.001 *
DM								
Without	Reference				Reference			
With	1.798	1.546	1.986	<0.001 *	1.714	1.512	1.888	<0.001 *
HTN								
Without	Reference				Reference			
With	2.068	1.573	2.676	<0.001 *	1.976	1.501	2.630	<0.001 *
Hyperlipidemia								
Without	Reference				Reference			
With	1.234	1.004	1.308	0.046 *	0.819	0.478	1.335	0.586
IHD								
Without	Reference				Reference			
With	1.462	1.297	1.682	<0.001 *	1.369	1.194	1.617	<0.001 *
CVD								
Without	Reference				Reference			
With	1.350	1.104	1.553	<0.001 *	1.271	1.043	1.495	0.007 *
Renal disease								
Without	Reference				Reference			
With	1.668	1.321	2.095	<0.001 *	1.609	1.225	1.962	<0.001 *
Tumor								
Without	Reference				Reference			
With	1.032	0.862	1.345	0.184	1.031	0.860	1.352	0.176
MetS								
Without	Reference				Reference			
With	2.065	1.482	2.798	<0.001 *	1.531	1.045	2.088	0.003 *
Hypercoagulable state								
Without	Reference				Reference			
With	1.526	0.862	2.060	0.289	1.306	0.671	1.884	0.462
Ischemic stroke								
Without	Reference				Reference			
With	1.398	1.167	1.592	<0.001 *	1.288	1.050	1.516	0.001 *
Cataract								
Without	Reference				Reference			
With	1.411	1.086	1.702	0.004 *	1.352	1.015	1.670	0.032 *
Glaucoma								
Without	Reference				Reference			
With	1.525	1.126	1.978	<0.001 *	1.488	1.034	1.826	0.018 *
Diabetic retinopathy								
Without	Reference				Reference			
With	1.279	1.089	1.465	<0.001 *	1.186	1.003	1.395	0.046 *
AMD								
Without	Reference				Reference			
With	1.382	0.979	1.891	0.072	1.297	0.875	1.742	0.088
CCI_R	1.234	1.125	1.290	<0.001 *	1.200	1.093	1.248	<0.001 *

* indicates a significant difference between the two groups with/without statin usage, *p* < 0.05. a presented as mean ± SD. CCI: Charlson comorbidity index, DM: diabetes mellitus, HTN: hypertension, IHD: ischemic heart disease, CVD: cardiovascular disease, MetS: metabolic syndrome, AMD: age-related macular degeneration.

**Table 3:** Risk for retinal vascular occlusion subgroups by Cox regression analysis.

Statin	With			Without (Reference)			With vs. Without (Reference)			
Retinal Vascular Occlusion Subgroups	Events	PYs	Rate (per 10^5^ PYs)	Events	PYs	Rate (per 10^5^ PYs)	Adjusted HR	95% CI	95% CI	*p*
Overall	124	413,862.59	29.96	486	1,235,055.74	39.35	0.704	0.591	0.873	<0.001 *
Central Retinal Artery Occlusion	31	413,862.59	7.49	126	1,235,055.74	10.20	0.679	0.564	0.841	<0.001 *
Arterial Branch Occlusion	27	413,862.59	6.52	119	1,235,055.74	9.64	0.620	0.518	0.779	<0.001 *
Central Retinal Vein Occlusion	34	413,862.59	8.22	124	1,235,055.74	10.04	0.758	0.640	0.952	0.008 *
Branch Retinal Vein Occlusion	32	413,862.59	7.73	117	1,235,055.74	9.47	0.743	0.629	0.939	<0.001 *

* indicates a significant difference between the two groups with/without statin usage, *p* < 0.05. a presented as mean ± SD. PYs = Person-years; Adjusted HR = Adjusted Hazard ratio: Adjusted for the variables listed in Table 2; CI = confidence interval.

## Data Availability

Data are contained within the article and Supplementary Materials.

## Supplementary Materials

The following are available online at https://www.mdpi.com/article/10.3390/ijerph18189864/s1, Table S1: Abbreviation, ICD-9-CM, and definition; Table S2: Years of follow-up.
