# Supplementary material for: Association between Statins and Retinal Vascular Occlusion: A Population-Based Cohort Study
Source: Int J Environ Res Public Health. 2021 Sep 18;18(18):9864. doi: 10.3390/ijerph18189864 (PMC8471930; doi:10.3390/ijerph18189864)
Supplement: Supplementary file 1 [file ijerph-18-09864-s001.zip › ijerph-1339756-supplementary.pdf]

Supplementary Materials

Association between Statins and Retinal Vascular Occlusion: A Population-Based Cohort Study

Chien-Cheng Chien 1 , Po-Huang Chen 2, Chi-Hsiang Chung 3,4,5, Chien-An Sun 6,7,
Wu-Chien Chien 3,4,5,8, Ke-Hung Chien 1

Contents

Table S1. Abbreviation, ICD-9-CM, and definition

Table S2. Years of follow-up

Table S1. Abbreviation, ICD-9-CM, and definition

	Abbreviation	ICD-9-CM / definition
**Events:**		
Retinal vascular occlusion		362.31–362.32, 362.35–362.36
Central retinal artery occlusion		362.31
Arterial branch occlusion		362.32
Central retinal vein occlusion		362.35
Branch retinal vein occlusion		362.36
**Complications:**		The time point is at the event onset and after the events
Macular edema		362.54
Neovascular glaucoma.		365.63
Cerebral infarction		433–434
**Comorbidities:**		
Diabetes mellitus	DM	250
Hypertension	HTN	401–405
Hyperlipidemia		272.0–272.4
Ischemic heart disease	IHD	410–414
Cerebrovascular disease	CVD	430–432, 435–437
Renal disease		580–589
Tumor		140–208
Metabolic syndrome	MetS	277.7
Hypercoagulable state		289.1–289.2
Ischemic stroke		433–434, 436, 852–853
Cataract		366

Glaucoma		365
Diabetic retinopathy		362.01–362.07
Age-related macular degeneration	AMD	362.51–362.52, 362.57
**Charlson comorbidity index revised**	CCI_R	CCI removed DM, HTN, IHD, CVD, renal disease, tumor, and ischemic stroke

Table S2. Years of follow-up

Statin	Min	Median	Max	mean ± SD
With	0.25	13.36	14.00	12.92 ± 1.61
Without	0.25	13.65	14.00	12.86 ± 1.97
Overall	0.25	13.59	14.00	12.87 ± 1.88
